# Prevalence of 13 polyomaviruses in actinic keratosis and matched healthy skin samples of immunocompetent individuals

**DOI:** 10.1186/s13027-022-00472-w

**Published:** 2022-12-01

**Authors:** Maria Gabriella Donà, Tarik Gheit, Maria Vincenza Chiantore, Maria Fenicia Vescio, Fabiola Luzi, Francesca Rollo, Luisa Accardi, Carlo Cota, Luisa Galati, Giovanna Romeo, Massimo Giuliani, Massimo Tommasino, Paola Di Bonito

**Affiliations:** 1 STI/HIV Unit, San Gallicano Dermatological Institute IRCCS, Rome, Italy; 2 International Agency for Research on Cancer, World Health Organization, Lyon, France; 3 EVOR Unit, Department of Infectious Diseases, Istituto Superiore di Sanità, Rome, Italy; 4 Epidemiology Unit, Department of Infectious Diseases, Istituto Superiore di Sanità, Rome, Italy; 5 Plastic and Reconstructive Surgery, San Gallicano Dermatologic Institute IRCCS, Rome, Italy; 6 Pathology Department, Regina Elena National Cancer Institute, IRCCS, Rome, Italy; 7 Department of Dermopathology, San Gallicano Dermatological Institute IRCCS, Rome, Italy; 8 Department of Medico-Surgical Sciences and Biotechnologies, Sapienza University of Rome-Polo Pontino, Latina, Italy; 9 IRCCS Istituto Tumori “Giovanni Paolo II”, Bari, Italy

**Keywords:** Polyomaviruses, Luminex-based HPyV assay, Actinic keratosis, Oncoviruses

## Abstract

**Background:**

Actinic keratosis (AK) is a precursor of cutaneous squamous cell carcinoma (cSCC). UV radiation is the major risk factor for AK, but certain human papillomaviruses (HPVs) of the beta genus are also involved in its development. Differently, the role of polyomaviruses (PyVs) in skin carcinogenesis is still debated. Fiftheen PyVs have been isolated from human tissues so far, including Merkel cell polyomavirus (MCPyV), the aetiological agent of Merkel cell carcinoma.

**Methods:**

The presence of 13 PyVs was assessed in skin samples from AK patients (n = 342). Matched fresh-frozen scrapings from healthy skin (HS) and AK lesions from 242 patients, and formalin-fixed paraffin-embedded AK biopsies from a different cohort of 100 patients were analyzed by multiplex PyVs genotyping assay.

**Results:**

The most frequent lesion site was the scalp in men (27.3%), and the cheek area in women (29.0%). Differences between men and women were significant for the scalp, the cheek area and the lips. Almost all the scrapings were PyV-positive (HS: 89.7%, AK: 94.6%; *p* = 0.04). The three most frequent PyVs were MCPyV, HPyV6 and JCPyV (HS: 87.2%, 58.7%, 6.6%, respectively; AK: 88.8%, 51.2%, 9.9%, respectively). HPyV9, TSPyV, BKPyV, HPyV7, LIPyV and SV40 were detected in < 2% of the scrapings. In most cases, matched HS and AK scrapings were both positive (MCPyV: 78.1%, HPyV6: 41.7%), or both negative for the individual genotypes (for the remaining PyVs). PyV prevalence in AK biopsies was 22.0%. Only MCPyV (21.0%) and HPyV6 (3.0%) were detected in these samples.

**Conclusions:**

PyV prevalence in HS and AK scrapings was high, but detection of PyVs exclusively in AK scrapings was rare. PyV positivity rate in AK biopsies was modest. Further research is need to reach firm conclusions regarding the role of these viruses in AK development.

## Introduction

Ultraviolet light is a key risk factor for cutaneous squamous cell carcinoma (cSCC) [1], but viral infections are also involved. Indeed, an association between beta-human papillomaviruses (HPVs) and cSCC has been found [2]. This neoplasia may be preceded by a precursor lesion, namely actinic keratosis (AK), which shows up to a 20% risk of progression to invasive cSCC [3]. The role of human polyomaviruses (HPyV) in skin carcinogenesis has been also examined, but with controversial results [4–7]. To date, 15 PyVs have been isolated from human specimens [8].

Worldwide studies have shown that HPyVs are common in healthy individuals [9]. Seroprevalence ranges from 23% (HPyV12) up to 90% for BK-, JC-, KI-, WU-, TSPyV, and HPyV10. For some HPyVs, i.e. MCPyV, HPyV6, HPyV7 and TSPyV, seroprevalence increases with age, whereas it remains constant for others (i.e., BKPyV and HPyV9) [10, 11]. A low seroprevalence has been reported for NJPyV, LIPyV and HPyV9 and SV40 while serology for HPyV14 and QPyV is not available yet. Serology studies have also shown multiple HPyV infections [12, 13].

Most HPyVs cause asymptomatic and self-limiting infections in immunocompetent hosts, while they are responsible for chronic conditions and severe diseases in oncological patients and organ transplant recipients (OTR). MCPyV is the causative agent for the majority of Merkel Cell Carcinomas (MCC), an aggressive neuroendocrine carcinoma of the skin [14]. Epidemiological studies reported an association of JCPyV, BKPyV, MCPyV and TSPyV with several malignancies [4, 15] and molecular studies showed viral integration and/or large T antigen (LTAg) expression in tumour samples [16–20]. For these reasons, these four viruses have been classified by the International Agency for Research on Cancer (IARC) as possible human carcinogens [21].

To further evaluate the role of PyVs in skin carcinogenesis, we have analyzed paired fresh-frozen scrapings from AK and healthy skin (HS) of immunocompetent AK patients, to evaluate the presence of both PyVs suspected to be oncogenic and those not yet associated with any pathology. Formalin-fixed paraffin-embedded (FFPE) AK biopsies were also analyzed.

## Material and methods

### Study populations

Two study groups were included: (1) patients with a clinical diagnosis of AK and a single lesion eligible for laser surgery, enrolled at the National Institute for Health, Migration and Poverty (NIHMP), Rome, Italy [22]; (2) patients with a histologically-confirmed diagnosis of AK performed at the San Gallicano Dermatological Institute (ISG), Rome, Italy. The socio-demographic data and lesion site were recorded. Informed consent was obtained from all the subjects enrolled at NIHMP. The patients enrolled at ISG provided a general consent for research use of surplus tissue at the moment of biopsy collection.

The study was approved by the Ethics Committee of the NIHMP (2014) and the San Gallicano Dermatological Institute (CE943/17; RS/1090/18).

### Skin scraping collection

Skin samples (n = 242) were collected by scraping the AK lesion with a sterile spatula without reaching the dermis. A scraping of healthy-looking skin (n = 242, hereafter HS) was also collected from the glabellar area using a different sterile spatula. All samples were stored until processing at − 80 °C.

### AK biopsies

FFPE skin biopsies (n = 197) from AK patients consecutively diagnosed at ISG in 2017 were retrieved from the archive of the Pathology Department. Only samples with sufficient material for the molecular analysis and confirmed AK diagnosis after hematoxylin and eosin (H&E) staining were included in this study. Upon visual inspection of the FFPE blocks, 95 were discarded because of the very small size of the tissue sample, and 102 cases were selected. From each of these FFPE blocks, the following sections were obtained: (1) 1 × 5 μm up to 5 × 5 μm sections, depending on the tissue size, for the PyVs typing by Luminex molecular assay; and (2) 1 × 2 μm section, used for H&E staining and confirmation of the histological diagnosis, performed by an expert dermatopathologist. Strict conditions during sectioning procedures were adopted in order to avoid cross-contamination between samples [23].

### DNA purification

Scrapings were thawed in ice and digested with proteinase K at 50 mAU/ml (Qiagen) in 0.4 ml of 20 mM Tris–HCl, 1 mM EDTA, 0.5% SDS pH 7.5, for 4 h at 50 °C. After proteinase K inactivation a 95 °C, the DNA was extracted using the Nuclisens Easymag automated platform according the manufacturer’s instructions. FFPE sections were incubated overnight at 37 °C in 0.25 ml of 10 mM Tris/HCl at pH 7.4, 0.5 mg/ml proteinase K, and 0.4% Tween 20. Then, to inactivate the proteinase K and to separate paraffin from the aqueous phase, samples were incubated at 95 °C for 10 min, centrifuged, and chilled on ice. The aqueous phase containing DNA was transferred to a new tube. Strict procedures were employed to avoid cross-contamination during DNA purification.

### Multiplex PyVs genotyping by beads-based Luminex assay

Identification of HPyV6, HPyV7, MCPyV, JCPyV, BKPyV, KIPyV, WUPyV, TSPyV, HPyV9, HPyV10, HPyV12, LIPyV and SV40 was performed using a highly sensitive type-specific multiplex assay, which also provides a semi-quantitative measure of the viral load. The IARC Multiplex PyVs molecular assay has been extensively validated [24, 25].

In each PCR-multiplex reaction, 10 µl of DNA was used, together with type-specific PyV primers and primers targeting β-globin gene, used as an internal control [26]. The detection limit of this multiplex PCR genotyping assay is 10 copies of viral genome [27]. The results are expressed as median fluorescence intensity (MFI) of at least 100 beads per bead set. For each probe, cut-off for positivity was computed as described previously [26]. The following negative controls were included in the assay: (1) H_2_O processed along with the samples to monitor DNA extraction and Luminex analysis and (2) H_2_O used as a negative control in multiplex PCR.

### Statistical analysis

Descriptive statistics were used to summarize the characteristics of the study populations. The overall and type-specific prevalence of PyVs in HS versus AK scrapings (unpaired groups) were compared using Chi-square tests.

The heatmap showing the results for matched HS and AK pairs was generated with the ClusVis program in order to show each patient status for each PyV (negative/positive) simultaneously in HS and corresponding AK scraping. When the paired HS-AK scrapings were both positive for a specific PyV, scraping pairs were also compared in terms of MFI value, a semi-quantitative measure of the viral load. To investigate possible determinants of positivity for any PyV, MCPyV, HPyV6, and JCPyV, univariate analyses were performed taking into account the following variables: age and sex for HS samples; age, sex and lesion site (sun-exposed: scalp, forehead, cheek, nose, auricle, lips; unexposed sites: arms, chest and torso) for AK samples. A *p* value < 0.05 was considered as statistically significant. The analyses were performed using MedCalc® Statistical Software version 20.014 (MedCalc Software Ltd, Ostend, Belgium; https://www.medcalc.org; 2021).

## Results

### Study population

Skin scrapings were collected from 242 immunocompetent patients (age range: 48–94 years; median age: 74, IQR: 68–80), of which 141 men (58.3%; median age: 76 years, IQR: 70–81) and 101 women (41.7%; median age: 72 years, IQR: 68–80). Most of the AK lesions were in the head region (n = 215, 88.8%). The lesions were significantly more common in the scalp for males (*p* < 0.0001), and in the cheek area (*p* = 0.0001), nose (*p* = 0.002) and chest (*p* = 0.006) for females (Table 1).

**Table 1:** Anatomic site distribution of the AK lesions in patients with fresh-frozen cytological (N = 242) and FFPE histological samples (N = 100)

AK site	AK patients cytological samples			AK patients histological samples		
	N = 242			N = 100		
	Male	Female	*p* value^a^	Male	Female	*p* value^a^
	N = 141	N = 101		N = 55	N = 45	
	n (%)	n (%)		n (%)	n (%)	
*Head*						
Scalp	71 (50.4)	3 (3.0)	**< 0.0001**	15 (27.3)	1 (2.2)	**0.0007**
Forehead	20 (14.2)	21 (20.8)	0.16	7 (12.7)	6 (13.3)	0.93
Cheeks	14 (10.0)	34 (33.7)	**0.0001**	5 (9.1)	13 (29.0)	**0.01**
Temples	15 (10.7)	7 (6.9)	0.32	7 (12.7)	1 (2.2)	0.06
Nose	6 (4.2)	16 (15.8)	**0.002**	6 (10.9)	6 (13.3)	0.7
Auricles	3 (2.1)	2 (2.0)	0.96	3 (5.5)	0 (0.0)	0.11
Eyes	0 (0.0)	0 (0.0)	0 (0.0)	2 (3.6)	2 (4.4)	0.84
Lips	3 (2.1)	0 (0.0)	0.14	0 (0.0)	4 (8.9)	**0.02**
*Trunk*						
Chest	2 (1.4)	9 (8.9)	**0.006**	3 (5.5)	3 (6.7)	0.80
Torso	4 (2.8)	7 (6.9)	0.49	4 (7.3)	3 (6.7)	0.91
*Extremities*						
Arms	3 (2.1)	2 (2.0)	0.96	2 (3.6)	3 (6.7)	0.48
Hands	0 (0.0)	0 (0.0)	0 (0.0)	0 (0.0)	1 (2.2)	0.27
Legs	0 (0.0)	0 (0.0)	0 (0.0)	1 (1.8)	2 (4.4)	0.45

Significant differences are highlighted in bold

AK, actinic keratosis; FFPE, formalin-fixed paraffin-embedded

^a^*p* value for the comparison between AK lesion site in male and female patients

We also analyzed 100 AK biopsies (two were discarded because evaluation of H&E-stained sections did not confirm AK diagnosis) collected from the second group of immunocompetent patients (median age: 74, IQR: 66–59): 55 (55.0%) from male (median age: 76 years, IQR: 66–81) and 45 (45.0%) from female patients (median age: 74 years, IQR: 64–78). The most frequent lesion site was the scalp in men (27.3%), and the cheek area in women (29.0%) (Table 1). Differences between men and women were significant for the scalp, the cheek area and the lips.

### PyV genotyping by Luminex beads-based assay

#### PyV prevalence in HS and AK scrapings

The results of the Luminex assay for the scrapings are shown in Table 2.

**Table 2:** Overall and type-specific prevalence of PyVs in scrapings from healthy-looking skin (HS) and actinic keratosis (AK) of 242 AK patients

PyV type	PyV-positive		
	n (%)		
	HS	AK	*p* value^a^
	n = 242	n = 242	
Any PyV	217 (89.7)	229 (94.6)	**0.04**
MCPyV	211 (87.2)	215 (88.8)	0.58
HPyV6	142 (58.7)	124 (51.2)	0.10
JCPyV	16 (6.6)	24 (9.9)	0.19
HPyV9	4 (1.7)	2 (0.8)	0.41
TSPyV	4 (1.7)	2 (0.8)	0.41
BKPyV	0 (0.0)	6 (2.5)	**0.01**
HPyV7	1 (0.4)	1 (0.4)	1.00
LIPyV	1 (0.4)	1 (0.4)	1.00
SV40	1 (0.4)	1 (0.4)	1.00
HPyV10, HPyV12, KIPyV, WUPyV	0 (0.0)	0 (0.0)	n.e

Significant differences are highlighted in bold

n.e., not estimable

^a^*p* value for the comparison between scrapings from HS and AK lesions

At least one PyV was detected in 217/242 HS (89.7%) and 229/242 AK scrapings (94.6%), respectively (*p* = 0.04). None of the HS or AK samples harbored HPyV10, HPyV12, KI- and WUPyV. MCPyV was found in 211/242 HS (87.2%) and 215/242 AK scrapings (88.8%), with no significant difference (*p* = 0.58). HPyV6 represented the second most common genotype, being detected in 142/242 (58.7%) HS and 124/242 AK scrapings (51.2%), respectively (*p* = 0.10). BKPyV was exclusively detected in AK samples (6/242, 2.5%). Multiple infections were frequently observed both in HS (141/242, 58.3%) and AK scrapings (118/242, 48.8%), *p* = 0.036, with a maximum of four different PyVs detected in a single sample.

For HS scrapings, neither positivity for any PyV, nor that for MCPyV, HPyV6, and JCPyV, significantly changed according to age and sex (data not shown). Regarding AK scrapings, positivity for any PyV, as well as that for MCPyV and HPyV6, was not significantly different according to age, sex and lesion site (data not shown). Similar results were obtained for JCPyV regarding age and lesion site. Differently, for female patients, JCPyV prevalence in AK scrapings was significantly higher than in male subjects (14.9% vs. 6.4%, *p* = 0.03).

#### PyV prevalence in matched HS and AK scrapings

The results for the paired HS and AK scrapings from each patient were then evaluated. Four outcomes were possible: concordantly negative (negHS-negAK) or positive results (posHS-posAK), and discordant results (negHS-posAK or posHS-negAK). When the paired HS-AK scrapings were both positive for a specific PyV, the MFI values were also evaluated. Table 3 shows the results for the 242 matched pairs of scrapings, regarding both any PyV and the individual genotypes (those not detected in any of the samples were excluded).

**Table 3:** Overall and type-specific results for PyV detection in matched HS and AK pairs (HPyVs not detected in any samples are not shown)

	HS versus AK			
	N = 242 n (%)			
	neg/neg	neg/pos	pos/neg	pos/pos
Any PyV	1 (0.4)	24 (9.9)	12 (5.0)	205 (84.7)
MCPyV	5 (2.1)	26 (10.7)	22 (9.1)	189 (78.1)
HPyV6	77 (31.8)	23 (9.5)	41 (16.9)	101 (41.7)
JCPyV	208 (86.0)	18 (7.4)	10 (4.1)	6 (2.5)
HPyV9	238 (98.4)	0 (0.0)	2 (0.8)	2 (0.8)
TSPyV	236 (97.5)	2 (0.8)	4 (1.7)	0 (0.0)
BKPyV	236 (97.5)	6 (2.5)	0 (0.0)	0 (0.0)
HPyV7	240 (99.2)	1 (0.4)	1 (0.4)	0 (0.0)
LiPyV	241 (99.6)	0 (0.0)	0 (0.0)	1 (0.4)
SV40	240 (99.2)	1 (0.4)	1 (0.4)	0 (0.0)

Four possible results were evaluated for paired HS-AK scrapings: concordant (neg/neg: both HS and AK negative; pos/pos: both HS and AK positive); discordant (neg/pos: negative HS/positive AK; pos/neg: positive HS/negative AK)

**Fig. 1:**
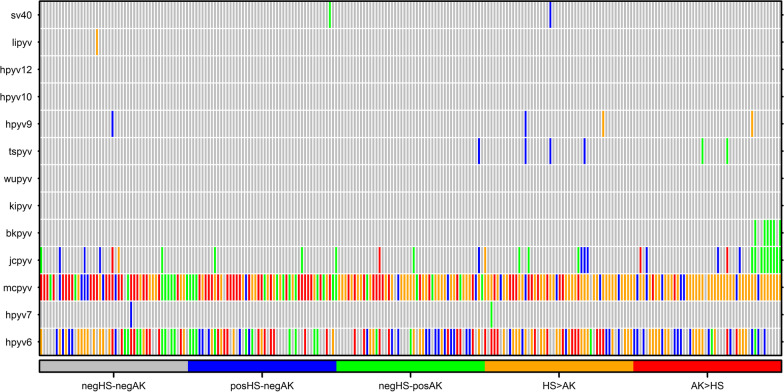
Heatmap for the comparison between healthy skin (HS) and actinic keratosis (AK) samples for each single PyV at individual level. Colors represent: (1) patients with both HS and AK samples negative for any PyV (negHS-negAK, grey); (2) patients with positive HS and negative AK for the virus indicated (posHS-negAK, blue); (3) patients with negative HS and positive AK for the virus indicated (negHS-posAK, green); (4) patients with both HS and AK positive for the virus indicated and showing a higher MFI value in HS than AK (HS > AK, orange), (5) patients with both HS and AK positive for the virus indicated and showing a higher MFI value in AK than HS (AK > HS, red)

A heatmap showing paired HS-AK results was also generated (Fig. 1). The most frequent finding for the paired scrapings was double positivity for MCPyV and HPyV6 (78.1% and 41.7%, respectively), and double negativity for JCPyV (86.0%). MCPyV and HPyV6 MFI values were higher in HS than matched AK scrapings in the majority of the concordantly positive cases (63.0% and 58.4%, respectively; data not shown) (Fig. 1). Conversely, for JCPyV double positive cases (2.5%), MFI value was higher in AK than HS scrapings.

#### PyV prevalence in FFPE AK biopsies

Overall PyV prevalence in AK biopsies was 22.0%. Specifically, 21 cases (21.0%) were positive for MCPyV and 3 cases (3.0%) for HPyV6 (2 samples harbored both MCPyV and HPyV6). None of the other HPyVs was detected in these specimens.

## Discussion

The role of HPyVs in human carcinogenesis has been well established for MCPyV [28]. Data are more controversial for other HPyVs. Epidemiological studies have suggested involvement in colon and brain cancer for JCPyV, thymic epithelial tumours for HPyV7 and prostate cancers for BKPyV. In experimental models, JCPyV and BKPyV also showed cellular transforming activities, mainly mediated by LTAg and Small T antigen (STAg) [4, 15, 29].

In this study, overall PyV prevalence in 242 AK patients was approximately 90% in both HS and AK scrapings. MCPyV was the most prevalent virus in both types of scrapings (almost 90%). Our findings are in line with the available data [30]. In fact, MCPyV is an ubiquitous virus that is detectable on the skin of healthy adults, although it has been also found in respiratory, urine, blood and anogenital samples [14]. Neither age nor sex significantly affected MCPyV prevalence. Conversely, previous findings reported an increased MCPyV positivity with the increasing of age [31, 32]. Of note, the median age of our patients was > 70 years, and MCPyV prevalence was already very high. Therefore, lack of significant association with age is to be expected.

HPyV6 was the second most frequent PyV in the scrapings, being detected in over 50% of the samples. HPyV6 has been found in several skin disorders, such as keratoacanthomas, basal and squamous cell carcinomas, as well as in healthy controls (12–30%) [9, 27, 33–37]. However, from the few published studies, there is a large difference in HPyV6 prevalence, and whether this virus plays a direct role in skin disorders is still unclear.

The third most frequent PyV was JCPyV (7–10%), the causative agent of progressive multifocal leukoencephalopathy, a fatal central nervous system disease. Interestingly, JCPyV prevalence in AK lesions of female patients was more than twofold higher than that of male patients, whereas there was no significant difference in JCPyV prevalence in HS samples according to sex. This observation deserves further investigation. Seroprevalence for JCPyV in immunocompetent adults is quite high (> 50%), but its presence in skin samples has not been frequently reported. A study that analyzed normal skin swabs from males failed to detect prevalent and incident JCPyV, further confirming that the skin is not the preferential site for its replication [27, 38].

HPyV9, TSPyV, HPyV7, BKPyV, LiPyV and SV40 were rarely detected. Their prevalence was indeed extremely low compared to the top three HPyVs. In addition, they were found only in AK scrapings but not in AK biopsies. These findings are consistent with the literature and suggest that the presence of some of these PyV in skin scrapings could be accidental contamination from other anatomical sites [39]. TSPyV is one of the etiological factors of a rare skin disorder observed in immunosuppressed patients (trichodysplasia spinulosa, TS). Previous studies failed to detect TSPyV-DNA in skin samples from healthy subjects [3, 27]. TSPyV does not seem to be associated with skin conditions other than TS. Indeed, it has not been detected either in skin malignancies or in a variety of inflammatory skin diseases [34].

Taking into account the three most commonly detected HPyVs in HS and AK scrapings, i.e., MCPyV, HPyV6, and JCPyV, no significant differences in their prevalence were observed when comparing the two types of specimens. In addition, when comparing matched HS-AK scrapings, PyV exclusive detection in the AK sample was rare (approximately 11% of the cases for MCPyV, and less frequently for the other PyVs). In the majority of cases, paired samples were both positive (for MCPyV and HPyV6) or both negative for the individual types (for the remaining PyVs). PyV prevalence in AK biopsies was rather modest. MCPyV was detected in 21% of the cases, in line with the results reported in another study, where a prevalence of 29% was reported [36]. A higher prevalence was found in AK cases from OTR (35.7%) [40]. A recent meta-analysis, which included six studies conducted on FFPE/fresh frozen AK sections, calculated a pooled prevalence rate for MCPyV of 6% [41], approximately half of that estimated for normal skin by the same meta-analysis (11%).

Interestingly, when comparing PyV prevalence in AK scrapings (around 95%) versus AK biopsies (22%), the difference we observed is comparable to that found in a study on HPV in AK. In fact, HPV prevalence decreased from 83% in AK swabs to 11% in the corresponding biopsies, obtained after stripping the surface of the lesion several times [42]. This seems to suggest that AK swabs/scrapings allow the detection of viruses present in the superficial layers of the skin, but this does not reflect their presence in AK lesional tissue.

In a previous work conducted on the same population as the present study, cutaneous HPVs with oncogenic capacity (e.g., HPV5, HPV8, HPV38) were detected in more than 50% of the AK scrapings [22]. Given the high prevalence of MCPyV and HPyV6 observed here, co-infections with cutaneous HPVs are frequent. Recently, it has been shown that MCPyV LTAg and STAg stimulate the transcriptional activity of the oncogenic HPV16 and HPV18 Long Control Region (LCR). Similar interactions between cutaneous HPVs and HPyVs have not been investigated but further studies are warranted in this regard [43].

A few limitations should be taken into account. AK biopsies were not collected from the same patients that provided AK scrapings, thus comparison between these types of samples needs to be regarded with caution. We did not analyze skin scrapings from healthy subjects, i.e., individuals not affected by AK. Finally, DNA integrity can be affected in FFPE samples, causing false negative results [23, 44], as also shown for HPV [23]. This may have caused an underestimation of PyV prevalence in AK biopsies. Conversely, the results obtained on skin scrapings are reliable, since these specimens were fresh-frozen at − 80 °C to ensure nucleic acid preservation and have been proven to be excellent for other molecular analyses [22, 45], thus representing a strength of the study. The very large sample size of AK represents an additional strength.

In conclusion, this study showed that PyV positivity rate in HS and AK scrapings collected from the same patients is very high, particularly for MCPyV and HPyV6. However, their prevalence in HS and AK scrapings did not significantly differ, and detection of PyV only in AK samples was rare when matched with the HS counterpart. In addition, PyV positivity rate in AK biopsies was modest. Based on our findings, we cannot draw firm conclusions regarding a possible role of PyVs in AK development. Further research is need to elucidate whether these viruses are mere bystanders or etiologically involved in AK.
